# Hikikomori syndrome: A cultural phenomenon not only limited to Japan

**DOI:** 10.1177/00207640231210113

**Published:** 2023-11-08

**Authors:** Inês Mateus Figueiredo

**Affiliations:** Clinic 3, Centro Hospitalar Psiquiátrico de Lisboa, Lisbon, Portugal

Hikikomori is a condition characterized by prolonged extreme social withdrawal with at least 6 months of duration, causing significant functional impairment or distress associated with this isolation. It comes from the verb *hiki*, which means to move back, and *komoru*, which means to come into. Hikikomori was formally recognized in Japan in the 1990s by Tamaki Saitō comparing it to an ‘adolescence without end’ turning into a serious public health problem in Asia (Fischborn, 2022).

It has been debated whether Hikikomori should be considered as a psychiatric disorder or a cultural syndrome and also, being the case of the former, whether it should be diagnosed if another psychiatric disorder can account for the symptoms. To help with the latter question, some authors suggest the term ‘secondary hikikomori’ if comorbidity is present and at least partially explains the syndrome, while in the absence of an intercurrent psychiatric diagnosis, the term ‘primary hikikomori’ should be used.

However originally suspected a result of sociocultural conditions specific to Japan, like Japan’s education system or economics, it is now seen in many countries with broad cultural differences (Eckardt, 2023).

Approximately 1% to 2% of adolescents and young adults suffer from Hikikomori in Asian. It mostly affects male patients (male:female 3:1) and the mean duration of social reclusion ranging, generally, from 1 to 4 years (Stip et al., 2016) but cases with more than 10 years are known.

Its etiology is not clear and several explanations have been proposed. Literature shows that dysfunctional family settings, traumatic experiences, introverted personality, and an ambivalent or avoidant attachment style can predispose to Hikikomori. Sociocultural factors, including a collapse of social cohesion, the creation of the Internet and subsequent changes to the way people interact with and within society, urbanization, globalization, and social mobility, may also contribute to development of Hikikomori (Stip et al., 2016).

Comorbidity with other psychiatric diagnosis, like schizophrenia, depression disorders, anxiety disorders, personality disorders, post-traumatic stress disorder and trauma-related disorders, adjustment disorders, and neurodevelopmental disorders, is very variable, ranging from practically half of cases to almost all cases (Teo & Gaw, 2010). An association with several suicidal risk factors and heavy internet is also common (Eckardt, 2023). Some people with certain physical illness can also isolate themselves until reach Hikikomori. Cases of great physical weakness and chronic pain disorders, some skin diseases and gastrointestinal diseases (irritable bowel syndrome, ulcerative colitis, and Crohn’s disease) can lead to social relationships avoidance (Kato et al., 2019).

Therapeutical approaches are generally psychotherapeutic but a multidisciplinary treatment with help of a team involving doctors, psychologists, nurses, and occupational therapists might lead to a better outcome. Treatment can include pharmacological options, especially for co-occurring psychiatric disorders, and family interventions (Kato et al., 2019).

Modern society, its technological advances and transformation social norms entangled with individual psychological factor allowed this syndrome to expand and to break the limits of a culture-bound phenomenon, so it is necessary to raise awareness to this problematic that is probably still a hidden epidemic in several countries in order to make a faster diagnosis and start optimal treatment to prevent the appearance of comorbidities or worsen it prognosis.
